# Normal Autophagic Activity in Macrophages from Mice Lacking Gα_i3_, AGS3, or RGS19

**DOI:** 10.1371/journal.pone.0081886

**Published:** 2013-11-28

**Authors:** Ali Vural, Travis J. McQuiston, Joe B. Blumer, Chung Park, Il-Young Hwang, Yolanda Williams-Bey, Chong-Shan Shi, Dzwokai Zach Ma, John H. Kehrl

**Affiliations:** 1 B-Cell Molecular Immunology Section, Laboratory of Immunoregulation, National Institute of Allergy and Infectious Diseases, National Institutes of Health, Bethesda, Maryland, United States of America; 2 Translational Mycology Unit, Laboratory of Clinical Infectious Disease, National Institute of Allergy and Infectious Diseases, National Institutes of Health, Bethesda, Maryland, United States of America; 3 Department of Cell and Molecular Pharmacology and Experimental Therapeutics, Medical University of South Carolina, Charleston, South Carolina, United States of America; 4 Department of Molecular, Cellular, and Developmental Biology, University of California Santa Barbara, Santa Barbara, California, United States of America; University of Illinois College of Medicine, United States of America

## Abstract

In macrophages autophagy assists antigen presentation, affects cytokine release, and promotes intracellular pathogen elimination. In some cells autophagy is modulated by a signaling pathway that employs Gα_i3_, Activator of G-protein Signaling-3 (AGS3/GPSM1), and Regulator of G-protein Signaling 19 (RGS19). As macrophages express each of these proteins, we tested their importance in regulating macrophage autophagy. We assessed LC3 processing and the formation of LC3 puncta in bone marrow derived macrophages prepared from wild type, Gnai3^-/-^, Gpsm1^-/-^, or Rgs19^-/-^ mice following amino acid starvation or Nigericin treatment. In addition, we evaluated rapamycin-induced autophagic proteolysis rates by long-lived protein degradation assays and anti-autophagic action after rapamycin induction in wild type, Gnai3^-/-^, and Gpsm1^-/-^ macrophages. In similar assays we compared macrophages treated or not with pertussis toxin, an inhibitor of GPCR (G-protein couple receptor) triggered Gα_i_ nucleotide exchange. Despite previous findings, the level of basal autophagy, autophagic induction, autophagic flux, autophagic degradation and the anti-autophagic action in macrophages that lacked Gα_i3_, AGS3, or RGS19; or had been treated with pertussis toxin, were similar to controls. These results indicate that while Gα_i_ signaling may impact autophagy in some cell types it does not in macrophages.

## Introduction

Macroautophagy (hereafter referred as autophagy) is an intracellular catabolic pathway providing cellular homeostasis. Autophagy facilitates bulk degradation and the recycling of mis-folded proteins, damaged organelles, and long-lived proteins [1]. Conserved protein kinases, lipid kinases, and ubiquitin-like protein-conjugation networks control autophagosome formation and cargo recruitment [2]. The autophagosome machinery interacts with cytoplasmic bulk material to be degraded and engulfs them to complete the maturation of autophagosomes, which eventually fuse with lysosomes. This forms autophagolysosomes leading to the degradation of the cytoplasmic constituents by lysosomal hydrolases [3]. Autophagy is a homeostatic cellular event, and unfavorable conditions such as starvation, growth factor deprivation, reduced cellular energy as well as various cell stressors such as oxidative stress, hypoxia, and certain chemicals can lead to the induction of autophagy [4]. In addition, bacterial toxins and intracellular infection by viruses or bacteria can also trigger the autophagic machinery as a means of cellular defense [5,6]. 

While some proteins are essential components of the autophagic process, others regulate the intracellular autophagic balance by affecting growth factor- and G-protein-mediated signaling pathways. Three such signaling proteins, G-protein inhibitory subunit 3 (Gα_i3_), and Activator of G-protein Signaling-3 (AGS3) and Regulator of G-protein Signaling 19 (RGS19) have been recognized as regulators of autophagy [7]. The initial association between autophagy and heterotrimeric G-protein signaling was reported using the human colonic carcinoma cell line HT-29, which constitutively degrades high mannose glycoproteins via an autophagic/lysosomal pathway. The treatment of HT-29 cells with pertussis toxin (PTX), which ADP-ribosylates heterotrimeric Gα_i_-proteins and prevents nucleotide exchange, reduced autophagic sequestration and restored the passage of N-linked glycoproteins through the Golgi complex [8]. Overexpression of wild type Gα_i3_ increased autophagic sequestration, whereas a GTPase deficient form inhibited it [8,9]. Consistent with its role in regulating autophagic sequestration, Gα_i3_ localized at the Golgi and endoplasmic reticulum as well as at the plasma membrane. In contrast, Gα_i2_ resided exclusively at the plasma membrane and its overexpression did not impact autophagy [10]. Also supporting a role for Gα_i3_ in autophagy regulation, overexpression of AGS3, a guanine nucleotide dissociation inhibitor (GDI) that stabilizes the GDP-bound conformation of Gα_i3_, resulted in enhanced autophagic sequestration [11,12]. Furthermore, RGS19, which augments the intrinsic GTPase activity of Gα_i3_, stimulated autophagy by favoring the GDP-bound conformation of Gα_i3_ [10,13]. Together, Gα_i3_, RGS19 and AGS3 reportedly controlled the cytoplasmic volume occupied by autophagic vesicles and regulated the flow through the exocytic and autophagic pathways [7]. Providing *in vivo* evidence for a role of Gα_i3_ in the regulation of autophagy, the lack of Gα_i3_ in starved primary mouse hepatocytes obviated the anti-autophagic effects of insulin and amino acids [14]. Moreover, a mechanistic explanation was proposed using HeLa cells as a model system. Nutrient deprivation recruited an AGS3-Gα_i3_ complex (GDP-bound state) to autophagic vesicles, whereas insulin stimulation led to Girdin/GIV triggered Gα_i3_ nucleotide exchange releasing Gα_i3_ from AGS3 and autophagosomes, thereby reversing the autophagic process [15]. 

The majority of our knowledge regarding the regulatory role of Gα_i3_ and its binding partners, AGS3 and RGS19, on autophagy has been generated using various cell lines and one study using primary mouse hepatocytes, where the mechanism that accounted for the impaired insulin or amino acid rescue of autophagy was not determined. Whether these proteins exhibit such regulatory roles in other cell types such as primary macrophages during inflammation or immune activation-induced autophagy is unknown. Using mice with targeted deletions of the genes encoding Gα_i3_, AGS3, or RGS19, we investigated autophagic induction/flux/recovery rates following nutrient deprivation, nigericin or rapamycin treatment of primary mouse macrophages. Our findings show that Gα_i_ nucleotide exchange; or the expression levels of Gα_i3_, AGS3, or RGS19 do not impact autophagy in these cells. 

## Material and Methods

### Ethics Statement

The animal experiments and protocols were performed according to the regulations of the National Institute of Allergy and Infectious Diseases (NIAID) Animal Care and Use Committee at the National Institutes of Health. The NIAID animal and care and use committee approved this study.

### Reagents

AGS3 antibody (polyclonal) was previously described [12]. Gα_i3_ anti-sera (polyclonal) was kindly provided Dr. Thomas Gettys (Pennington Biomedical Research Center, Baton Rouge, LA). The LC3B antibody (L7543), β-actin-peroxidase antibody (A3854), PTX from *Bordatella pertussis* (P7208), rapamycin from *Streptomyces hygroscopicus* (R0395), nigericin sodium salt (N7143) and phorbol 12-myristate 13-acetate (PMA) were purchased from Sigma-Aldrich. The other reagents were purchased as follows: GFP and Atg7 antibody (2555, 2631, respectively, Cell Signaling Technology), ubiquitin antibody (clone FK2; 04-263, Millipore), Gα_i2_ and p62 antibody (sc-7276, sc-10117, respectively, Santa Cruz Biotechnology), Bafilomycin A1 *Streptomyces griceus* (EMD Millipore, 196000) and Alexa Fluor 568 Goat Anti-Rabbit IgG (H+L) (Life Technologies, A11011). The following secondary antibodies were used for Western blot experiments and immunocytochemical blocking purposes, respectively: peroxidase-conjugated Donkey Anti-Rabbit IgG (Jackson ImmunoResearch Laboratories, 711-036-125); peroxidase-conjugated Donkey Anti-Mouse IgG (Jackson ImmunoResearch Laboratories, 715-036-150) and normal goat serum (Jackson ImmunoResearch Laboratories, 005-000-121). 

### Animals

Wild-type C57BL/6 mice were from Jackson Laboratory. AGS3-deficient (*Gpsm1-/-*) have been described [16]. The Gα_i3_-deficient (*Gnai3* ^*-/-*^) mice were kindly provided by Lutz Birnbaumer (National Institute of Environmental Health Sciences, National Institute of Health, Research Triangle Park, NC) and backcrossed ten times to C57BL/6. The *Rgs19* GFP knock-in mice were generated by Ozgene (Australia). The Atg7^-/-^ mice were kindly provided by Dr. Masaaki Komatsu (Tokyo, Japan). 

### Bone marrow-derived macrophage (BMDM) and THP-1 cell culture

Bone marrow cells were isolated from mouse femur and tibia. The cells were grown in RPMI media containing 10% (vol/vol) fetal bovine serum (FBS, Invitrogen) and 1% penicillin-streptomycin-amphotericin B (EMD Millipore, 516104) supplemented with 30% (vol/vol) conditioned medium obtained from the supernatant of L929 cells. The medium was changed every 2 days and macrophages were used after 7-10 days of culture [17]. THP-1 cells were obtained from American Type Culture Collection. THP-1 cells were maintained in RPMI-1640 medium containing 10% (vol/vol) FBS, 1% penicillin-streptomycin-amphotericin B and 50 μM 2-mercaptoethanol. THP-1 cells were differentiated into macrophages by treatment for 3 hours with 50 nM PMA.

### Immunoblotting

BMDM and PMA-differentiated THP-1 cells were seeded in 12-well plate at 8.0x10^5^-1.0x10^6^ cells/well 18 hours prior to collection and preparation of cell lysates. Following the appropriate treatments, BMDM cells were washed with cold PBS, and lysed with a buffer of 20 mM HEPES, pH 7.4, 50 mM β-glycerophosphate, 1% (vol/vol) Triton X-100, 2 mM EGTA (Ethylene glycol-bis-(2-aminoethyl)-N,N,N', N'-tetraacetic acid), 10% (vol/vol) glycerol and protease inhibitor cocktail (Roche Applied Sciences, 11836170001). Lysates were shaken on ice for 15-20 minutes followed by centrifugation at 13,000 rpm for 10 minutes at 4°C. 4Χ NuPAGE LDS sample buffer (Life Technologies, NP0008) was added to supernatant fraction and boiled for 10 min, and the samples resolved on 4-20% SDS-PAGE gels. The proteins were transferred onto a nitrocellulose membrane with the iBlot transfer apparatus (Invitrogen). The membrane was blocked in TBST solution containing 5% nonfat dry milk for 1 hour at room temperature. Following three 5-10 minute washes in TBST; the membrane was incubated at 4 °C with the indicated primary antibody overnight. The membrane was washed three times and incubated with HRP-conjugated secondary antibody for 1 hour. An enhanced chemiluminescence solution was added to analyze the protein expression levels. Blots were scanned and imported into Photoshop software (Adobe Systems). 

### Immunocytochemistry

BMDM and PMA-differentiated THP-1 cells were cultured on 35 mm glass bottom dishes (MatTek Corporation, P35G-0-10-C) for 18-24 hours prior to exposure to the indicated treatments. The cells were washed with cold PBS and fixed overnight with methanol at -20 °C. 4% normal goat serum was used to reduce non-specific binding to the cells for 1 hour at room temperature, and followed by overnight incubation at 4 °C with the indicated primary antibody. Cells were incubated at 37 °C with Alexa Fluor-conjugated secondary antibodies for 1 hour and visualized by confocal microscopy. To measure autophagy, either the endogenous LC3 or GFP-LC3 puncta (≥1 μm) were quantified in 50-100 cells per sample. 

### Flow cytometry

Single cells were re-suspended in PBS, 2% FBS and stained with fluorochrome-conjugated or biotinylated antibodies against B220 (BD Pharmingen, RA3-6B2), CD11c (BD Pharmingen, HL3), and Gr-1 (BD Pharmingen, RB6-8C5). Biotin-labeled antibodies were visualized with fluorochrome-conjugated streptavidin (eBioscience, 49-4317-80). LIVE/DEAD Fixable Aqua Dead Cell Stain Kit (Molecular Probes, L34957) was used in all experiments to exclude dead cells. Data acquisition was done on FACSCanto II (BD) flow cytometer and analyzed with FlowJo software (Tree Star).  

### Confocal microscopy and image preparation

The images were captured using PerkinElmer Ultraview spinning-wheel confocal system mounted on a Zeiss Axiovert 200 equipped with an argon-krypton laser with 100Χ Plan-Aprochromax oil-immersion objective (numerical aperture 1.4). Volocity 4.1 (PerkinElmer) and Adobe Photoshop 7 (Adobe Systems) were used to process the images. 

### Autophagic proteolysis of long-lived proteins

Autophagic proteolysis of long-lived proteins was assayed as previously described with minor modifications [18]. Briefly, 1x10^6^ bone marrow derived macrophages were plated in a well of a 12 well plate in L929-conditioned media and incubated at 37° C, 5% CO_2_ overnight. Media was removed and replaced with 1 ml complete media with 1μCi/ml ^3^H-valine and incubated at 37° C, 5% CO_2_ overnight. After 24 hours, the labeling media was removed and cells were washed once with 0.5ml/well of complete media. Following the wash, macrophages were incubated in complete media overnight (approximately 16 hours) at 37 °C, 5% CO_2_ to chase the labeled short lived proteins. The following day, macrophages destined for autophagy induction were washed with 1 ml complete media containing 50 μg/ml rapamycin and then incubated in 0.5 ml complete media containing 50 μg/ml rapamycin for 10 minutes at 37° C, 5% CO_2_ [19]. This 10-minute incubation step was repeated twice and then 0.5 ml complete media containing 50 μg/ml rapamycin was added. Assigned wells were washed and incubated as noted above with complete media containing 50 μg/ml rapamycin with the addition of wortmannin (1 μM). Control cells were washed with 1 ml complete media and then received 0.5 ml complete media for the assay. After two hours of incubation at 37° C, 5% CO_2_, 112 μl of tricholoracetic acid (TCA) was added to each well and cells were then scraped and collected. Cell lysates were vigorously pipetted, incubated on ice for 20 minutes, and centrifuged at 13,000 rpm for 5 minutes. The supernatant was removed and the amount of ^3^H levels measured using a scintillation counter. TCA-precipitated protein pellets were dissolved in 0.2M NaOH and measured for ^3^H levels using a scintillation counter. The percent protein degradation was calculated as the soluble radioactivity divided by the total radioactivity of the sample representing the soluble radioactivity plus the cellular radioactivity. 

### Statistical analysis

Data are expressed as a mean value ± SEM. All results were confirmed in at least three independent experiments. Data were analyzed using Student’s *t* test *or* ANOVA and *p* < 0.05 was considered statistically significant.

## Results

### The lack of Gα_i3_ or inhibition of Gα_i_ nucleotide exchange does not affect basal autophagy or autophagic induction/sequestration in primary mouse macrophages

**Figure 1:**
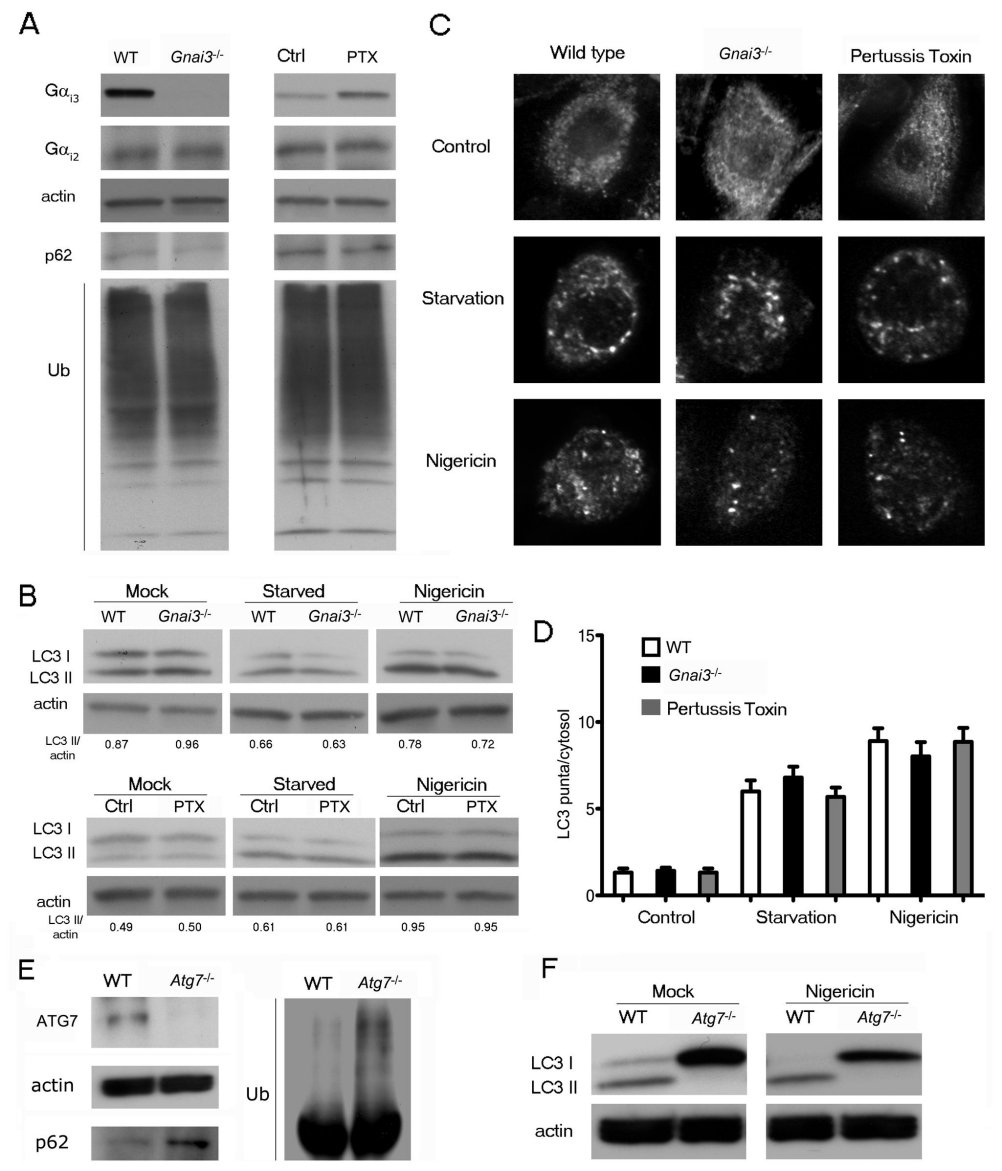
The lack of Gα_i3_ or PTX treatment does not affect autophagy induction in primary mouse macrophages. (*A*) Immunoblot analysis of cell lysates from BMDM prepared from wild type mice; Gnai3^-/-^ mice; or wild type mice pre-exposed to PTX (200 ng/ml for 2h), or not, to assess the steady state levels of Gα_i3_, Gα_i2_, p62 and ubiquitin proteins. (*B*) Immunoblot analysis of cell lysates from BMDM prepared from wild type mice; Gnai3^-/-^ mice; or wild type mice pre-exposed to PTX as above, or not, either not further manipulated, starved (HBSS for 1h with 100 nM Bafilomycin A1), or treated with nigericin (4 µM for 4h) to assess LC3-II/actin band ratios. (*C*) Endogenous LC3 immunocytochemistry of BMDM from wild type mice, Gnai3^-/-^ mice, or from wild type mice pre-exposed to PTX (200 ng/ml for 2h) either not further manipulated, starved (HBSS for 1h with 100 nM Bafilomycin A1), or treated with nigericin (4 µM for 4h). Representative images are shown for each condition. (*D*) Quantification of endogenous LC3 dots performed by fluorescence microscopy for at least 70-100 cells from experiment shown in part C. Data represents the mean LC3 puncta per cytosol ± SEM for three independent experiments for each condition. (*E*) Immunoblot analysis of cell lysates from BMDM prepared from wild type mice and Atg7^-/-^ mice to assess the steady state levels of p62 and ubiquitin proteins. (*F*) Immunoblot analysis of cell lysates from BMDM prepared from wild type mice and Atg7^-/-^ deficient mice, either not further manipulated, or treated with nigericin (4 µM for 4h) to assess LC3-II/actin band ratios.

We first verified that bone marrow derived macrophages (BMDM) from the Gnai3^-/-^ mice lacked Gα_i3_ and assessed the impact of the loss on the expression of Gα_i2_ as well as the effect of PTX treatment on the expression of both isoforms of Gα_i_. Like other hematopoietic cells macrophages express little or no Gα_i1_. As expected the Gnai3^-/-^ macrophages lacked Gα_i3_ and Gα_i2_ levels were not appreciably altered (Figure 1A). PTX treatment resulted in a modest increase in Gα_i3_, but not Gα_i2_ (Figure 1A). To check basal autophagy levels in BMDM we measured steady-state levels of autophagy marker protein SQSTM1/p62 and of ubiquitinated proteins since they increase following the suppression of basal autophagy [20]. We found similar levels of p62 expression and of ubiquitinated proteins in cell lysates prepared from wild type versus Gα_i3_-deficient BMDM; and in PTX treated versus control BMDM (Figure 1A). These results indicate that neither Gα_i3_ deficiency nor the inhibition of Gα_i_ nucleotide exchange with PTX results in a significant difference in steady-state autophagy levels in primary mouse BMDM. 

Next, we assessed steady-state autophagy and autophagic induction/sequestration via LC3 immunoblotting and quantification of LC3 puncta. The LC3 protein is found in cytosol as LC3-I form, which has a molecular weight of 18 kDa. Following autophagic induction, it becomes lipidated to autophagic vesicle membranes as the LC3-II form with a molecular weight of 16 kDa. Similar to the previous results assessing p62 and ubiquitinated proteins, we found that Gα_i3_ deficiency or PTX treatment did not alter the basal LC3-I/LC3-II ratio in cell lysates (Figure 1B). We induced autophagy by starvation or nigericin treatment of BMDM [21]. Starvation was performed in the presence of bafilomycin A1, which inhibits the fusion between autophagosomes and lysosomes to reveal the autophagic activation. As a complementary approach, nigericin challenge was performed to compare inflammatory response-dependent autophagy induction. Nigericin is a bacterial toxin and ionophore that raises the intracellular pH of lysosomes thereby inhibiting autophagosome-lysosome fusion [22]. In addition, it also activates NLRP3 inflammasome, which can trigger autophagosome formation [23,24]. We found that the Gα_i3_-deficient and the PTX-exposed macrophages responded similar to wild type or control macrophages following autophagy induction (Figure 1B). To confirm this result, we employed LC3 immunochemistry to measure the extent of induction/sequestration by imaging autophagic vesicles formation (Figure 1C). We found that the average number of autophagic puncta were similar for all groups suggesting that neither Gα_i3_ alone nor the ADP-ribosylated Gα_i_ subunits affected the induction/sequestration rates of autophagy in primary macrophages (Figure 1D). 

Similar experiments were performed with BMDM prepared from Atg7^-/-^ mice. We verified the lack of ATG7 expression in the BMDM derived from the Atg7^-/-^ mice (Figure 1E). In contrast to the previous experiments the steady-state levels of p62 and of ubiquitinated proteins were elevated in the Atg7^-/-^ BMDM (Figure 1E). Also, as expected nigericin treatment did not induce the appearance of LC3-II in the Atg7^-/-^ BMDM (Figure 1F). 

### The lack of AGS3 does not affect basal autophagy or autophagic induction/sequestration in primary mouse macrophages

**Figure 2:**
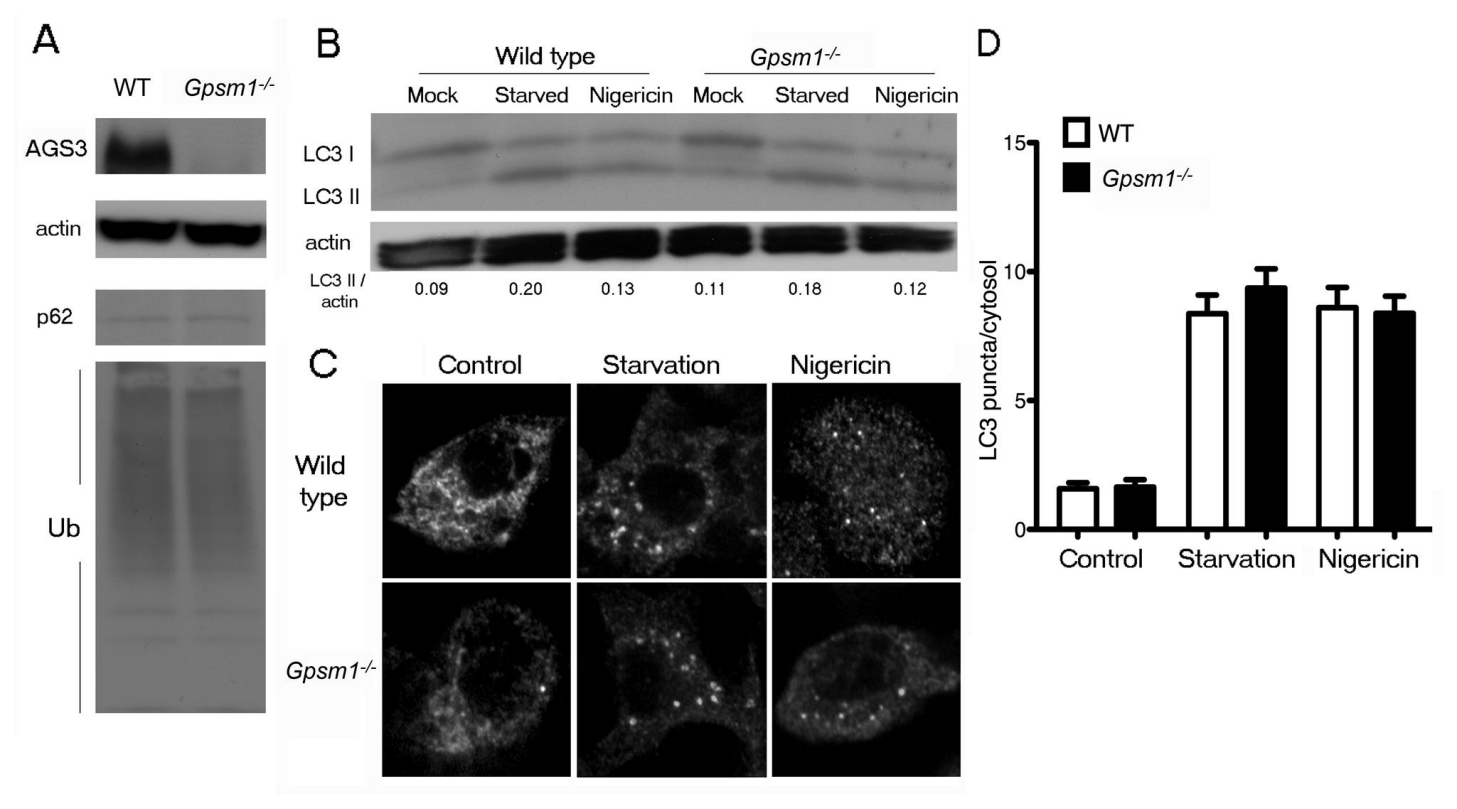
The lack of AGS3 does not affect autophagy induction in primary mouse macrophages. (*A*) Immunoblot analysis of cell lysates from BMDM prepared from wild type mice or Gpsm1^-/-^ mice to assess the steady state levels of Gα_i3_, Gα_i2_, p62 and ubiquitin proteins. (*B*) Immunoblot analysis of cell lysates from BMDM prepared from wild type mice or Gpsm1^-/-^ mice either not manipulated, starved (HBSS for 1h with 100 nM Bafilomycin A1), or treated with nigericin (4 µM for 4h) to assess LC3-II/actin band ratios. (*C*) Endogenous LC3 immunocytochemistry of BMDM from wild type mice or Gpsm1^-/-^ mice either left untreated, starved (HBSS for 1h with 100 nM Bafilomycin A1), or treated with nigericin (4 µM for 4 h). Representative images are shown for each condition. (*D*) Quantification of endogenous LC3 dots performed by fluorescence microscopy for at least 70-100 cells from experiment shown in part C. Data represents the mean LC3 puncta per cytosol ± SEM for three independent experiments for each condition.

AGS3 is a member of a family of proteins that contain GoLoco/GPR motifs [25,26]. It is a seventy-two kilodalton protein with a modular structure possessing seven tetratricopeptide repeats termed the TPR domain in its N-terminal portion, a linker domain, and 4 GoLoco/GPR motifs located in its C-terminal portion, each capable of binding GDP-Gα_i_ [27,28]. In HeLa cells AGS3 directly binds LC3 and recruits Gα_i3_ to LC3-positive membranes upon starvation, and thereby promotes autophagy by inhibiting the G protein [15]. However, mice lacking AGS3 exhibit a lean body habitus with a reduced fat mass, a result not readily explained by the overexpression studies [16]. More consistent with the *in vivo* phenotype, knock-down studies using HeLa cells, but in addition HEK 293 cells, revealed an increase in steady state LC3II levels and autophagic structures, which increased further in the presence of balifomycin A1 [12]. To determine whether the lack of AGS3 in macrophages affected basal or autophagic induction/sequestration we performed similar experiments as we did with the Gnai3^-/-^ mice. We first verified that mouse BMDM expressed AGS3 and the BMDM from Gpsm1^-/-^ mice lacked expression by immunoblotting cell lysates from wild type and mutant BMDM (Figure 2A). Next, we checked p62 and the amount of ubiquitinated proteins in lysates from wild type and AGS3 deficient BMDM. We found no significant difference (Figure 2A). In contrast to the knock-down studies in HeLa and HEK 293 cells, the BMDM had a similar basal level of LC3II. Starvation and nigericin treatment resulted in a similar increase in LC3 processing in the wild type and mutant BMDM (Figure 2B). In accord with this data the visualization and quantitation of the average number of autophagic puncta also appeared similar between wild type and AGS3 deficient BMDM (Figure 2C and 2D). Together, these results provide strong evidence that AGS3 does not have an essential regulatory role in autophagic induction/sequestration in BMDM.

### The lack of RGS19 does not affect basal autophagy or autophagic induction/sequestration in primary mouse macrophages

**Figure 3:**
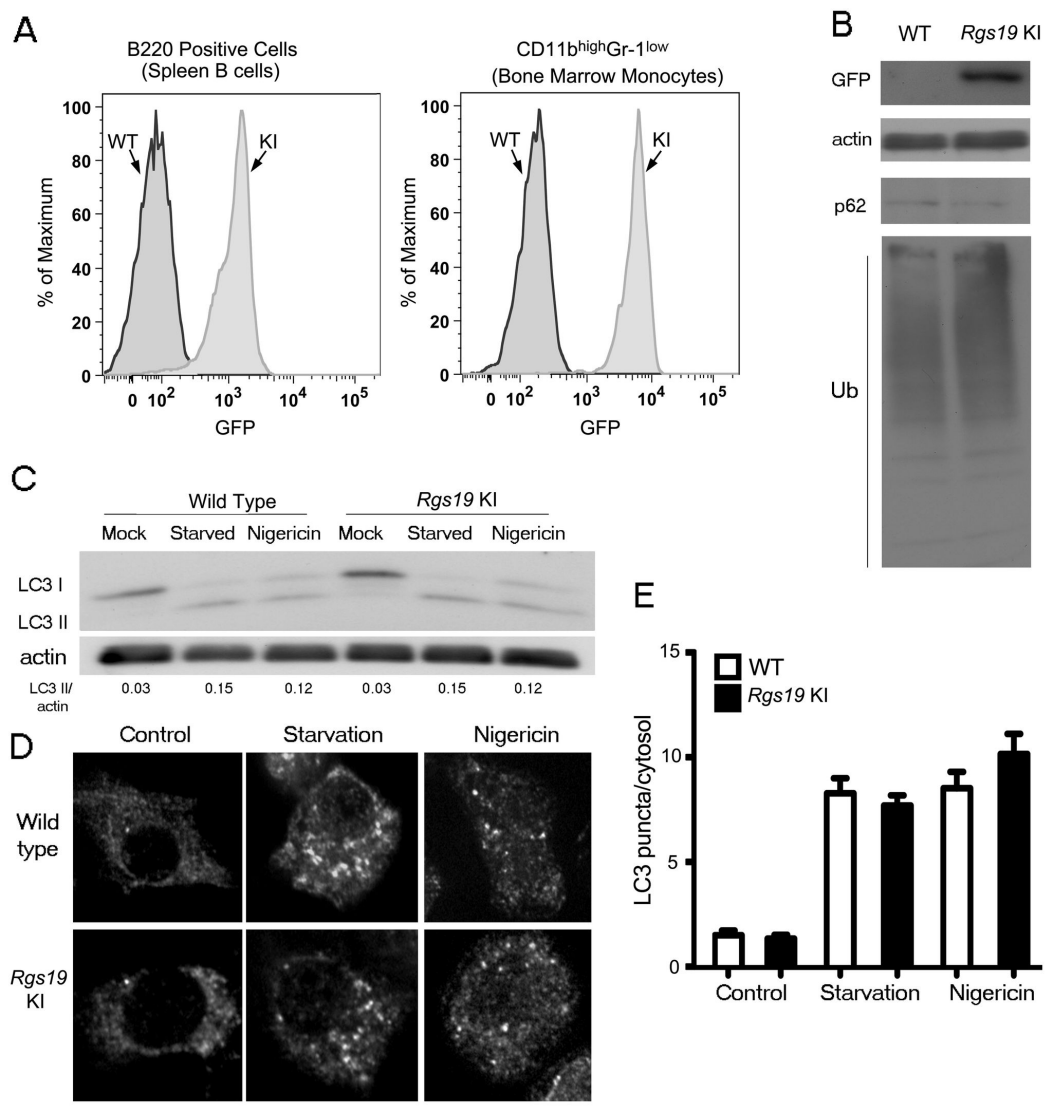
The lack of RGS19 does not affect autophagy induction in primary mouse macrophages. (*A*) Flow cytometry analysis of GFP expression in splenic B cells and bone marrow monocytes from *Rgs19* GFP KI mice. (*B*) Immunoblot analysis of cell lysates from BMDM prepared from wild type mice or *Rgs19* GFP KI mice to assess the steady state levels of Gα_i3_, Gα_i2_, p62 and ubiquitin proteins. (*C*) Immunoblot analysis of cell lysates from BMDM prepared from wild type mice or *Rgs19* GFP KI mice either not manipulated, starved (HBSS for 1 h with 100 nM Bafilomycin A1), or treated with nigericin (4 µM for 4h) to assess LC3-II/actin band ratios. (*D*) Endogenous LC3 immunocytochemistry of BMDM from wild type mice or *Rgs19* GFP KI mice either not manipulated, starved (HBSS for 1 hour with 100 nM Bafilomycin A1), or treated with nigericin (4 µM for 4 hours). Representative images are shown for each condition. (*E*) Quantification of endogenous LC3 dots performed by fluorescence microscopy for at least 70-100 cells from experiment shown in part C. Data represents the mean LC3 puncta per cytosol ± SEM for three independent experiments for each condition.

RGS19 has also been reported to regulate autophagy by favoring GDP-bound state of Gα_i3_ [13]. RGS19 is a member of the RGS protein family possessing an N-terminal rich cysteine region and a typical C-terminal RGS domain [29]. Like most other RGS protein family members it greatly enhances the intrinsic GTPase activity of Gα_i_ and Gα_q_ family members [29,30]. It has been previously localized at intracellular membrane sites in conjunction with Gα_i3_ [31]. RGS19 mRNA is predominately expressed in hematopoietic cells and is well expressed in lymphocytes and macrophages (IMMGEN GeneSkyline/Constellation Symbol = Rgs19). We have generated Rgs19^-/-^ mice using a GFP knock-in approach and have found strong GFP expression in hematopoietic cells (Figure 3A). These mice are born with normal Mendelian frequency and exhibit no gross abnormalities. The targeting resulted in the loss of Rgs19 mRNA in all tested cell types (Park, C., unpublished observation). Because of the lack of a RGS19 antibody suitable for immunoblotting endogenous protein, we have shown the expression of GFP by flow cytometry and in cell lysates by immunoblotting as a surrogate for the level of RGS19 expression in BMDM (Figure 3A & B). When compared to wild type mice the level of p62 and ubiquitinated proteins in the cell lysates of BMDM were unaltered (Figure 3B). In addition, the basal level of LC3 processing was similar in the mutant mice (Figure 3C). Finally the response to starvation and nigericin treatment was assessed. Again we could not detect a difference between wild type and RGS19 deficient BMDM (Figure 3 C-E). The lack of visible GFP expression in the *Rgs19* GFP KI BMDM is secondary to the fixation, which results in the loss of the GFP signal. Our results demonstrate that RGS19 does not have a major regulatory role in the control of autophagy induction/sequestration in BMDM.

### The lack of Gα_i3_, AGS3 or inhibition of Gα_i_ nucleotide exchange does not affect autophagic proteolysis in primary mouse macrophages

**Figure 4:**
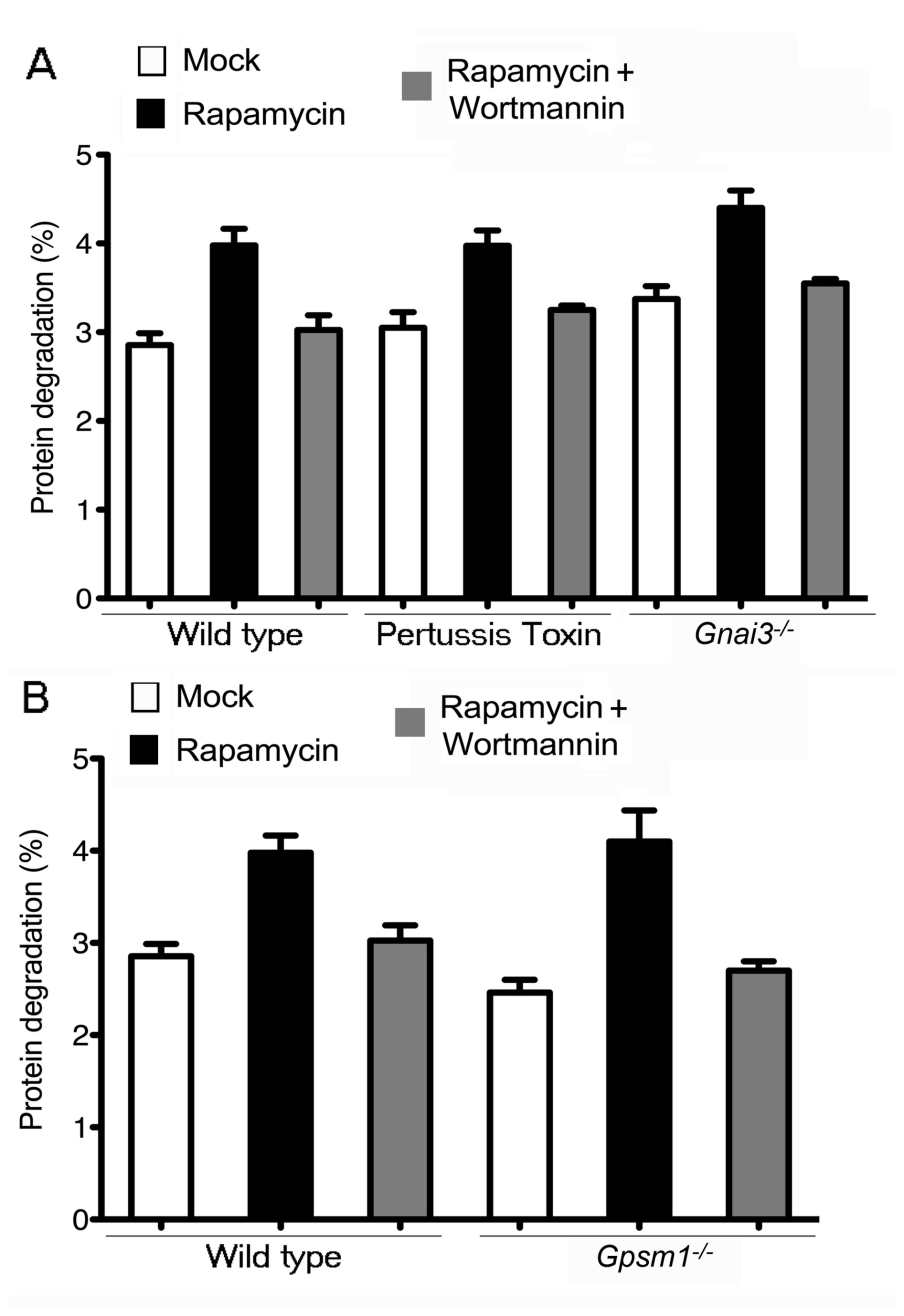
The lack of Gα_i3_, the lack of AGS3 or PTX treatment does not affect autophagic proteolysis in primary mouse macrophages. (*A*) The analysis of autophagic proteolysis in BMDM prepared from wild type mice; wild type mice pre-exposed to PTX (200 ng/ml for 2h); or Gnai3^-/-^ mice, either not further manipulated, treated with rapamycin (50 μg/ml for 2h), or treated with wortmannin (1 μM for 2h) and rapamycin (50 μg/ml for 2h) (*B*) The analysis of autophagic proteolysis in BMDM prepared from wild type mice; or Gpsm1^-/-^ mice, either not further manipulated, treated with rapamycin (50 μg/ml for 2h), or treated with wortmannin (1 μM for 2h) and rapamycin (50 μg/ml for 2h).

Having measured the steady-state autophagy and autophagic induction levels at static time points in BMDM, we concluded that neither PTX pre-treatment nor the lack of Gα_i3_, AGS3 and RGS19 affected the accumulation of autophagosomes by inducing autophagy or by impairing its completion. However, these results by themselves are insufficient to conclude that these proteins had no role in autophagic flux, which is defined as the complete process of autophagy including the delivery autophagic cargo to lysosomes for degradation [32]. Therefore, we assessed the autophagic levels in a more comprehensive and time course dependent manner by examining autophagic flux in the different macrophages. A standard method to determine the autophagic flux is to measure the turn-over of long-lived proteins [33]. Therefore, we induced autophagy in wild type, *Gnai3* ^*-/-*^, or *Gpsm1* ^*-/-*^ BMDM or in wild type BMDM previously treated with PTX. We then measured the increase in autophagic protein degradation rates. To verify that the rapamycin induced increase in the turn-over of long lived proteins depended upon autophagy, we added wortmanin, a phosphatidylinositol 3'-kinase inhibitor that impairs autophagic sequestration and protein degradation. Our results revealed no significant difference with regard to degradation rates of long-lived proteins in PTX pre-treated, *Gnai3* ^*-/-*^, or *Gpsm1* ^*-/-*^ BMDM suggesting that Gα_i3_ and AGS3 do not take a regulatory role during the entire course of the rapamycin-induced autophagic process in BMDM (Figure 4 A & B).

### The loss of Gα_i3_ or AGS3; or the inhibition of Gα_i_ nucleotide exchange did not affect anti-autophagic activity in primary mouse macrophages

**Figure 5:**
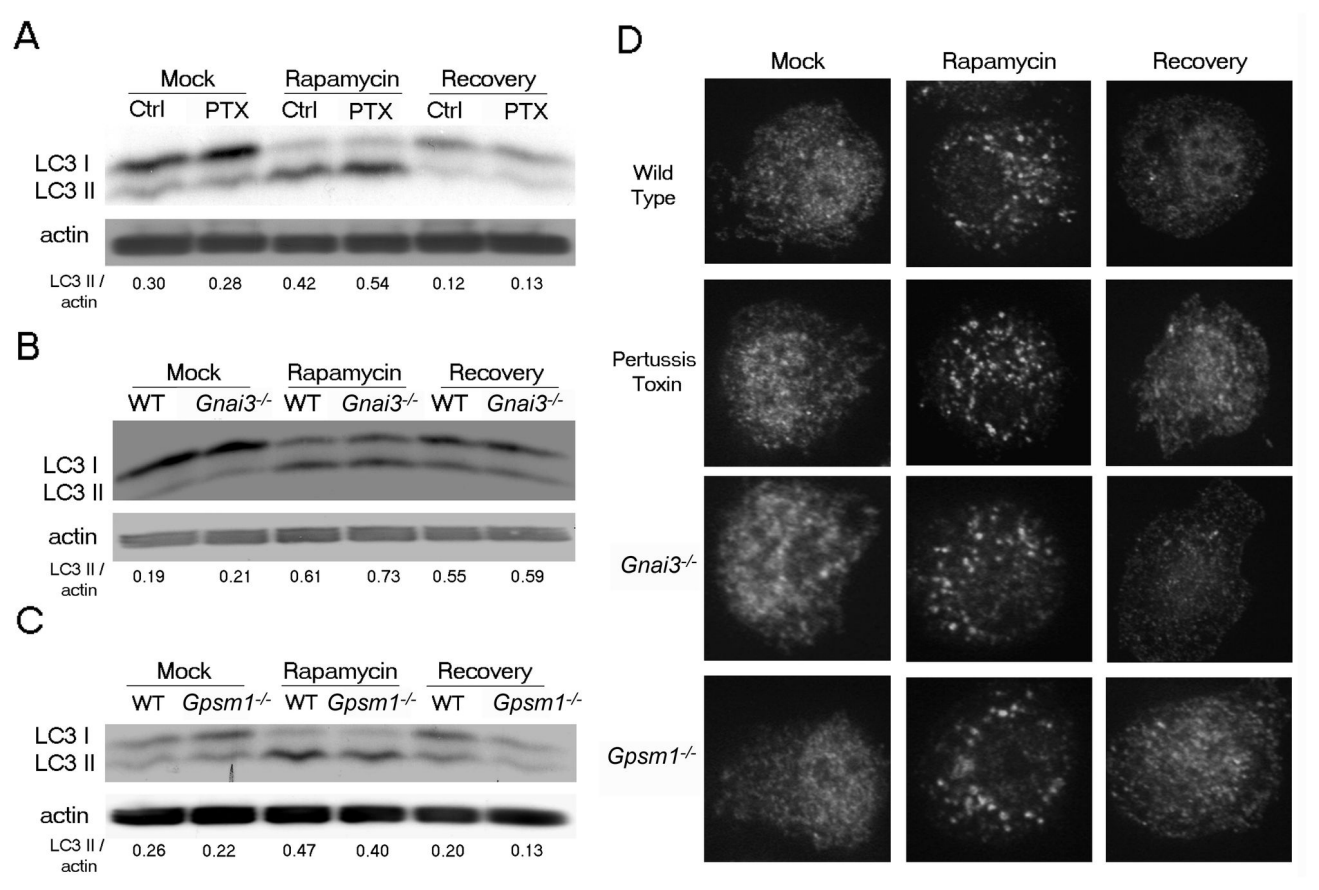
PTX treatment, the lack of Gα_i3,_ or the lack of AGS3 does not affect the anti-autophagic activity in primary mouse macrophages. (*A*) Immunoblot analysis of cell lysates from BMDM prepared from wild type mice; or wild type mice pre-exposed to PTX (*B*) Gnai3^-/-^ mice; (*C*) Gpsm1^-/-^ mice, either not further manipulated, treated with rapamycin (50 μg/ml for 2h), treated with complete media alone for 2-3 hours following rapamycin (50 μg/ml for 2h) treatment. (*D*) Endogenous LC3 immunocytochemistry of BMDM from wild type mice, wild type mice pre-exposed to PTX (200 ng/ml for 2h), Gnai3^-/-^ mice, and Gpsm1^-/-^ mice either not further manipulated, treated with rapamycin (50 μg/ml for 2h), or treated with complete media alone for 2-3 hours following rapamycin (50 μg/ml for 2h) treatment. Representative images are shown for each condition.

Anti-autophagic activity reverses the autophagic process, whereby autophagic vesicles disappear and autophagy-dependent degradation decreases. Since the anti-autophagic actions of insulin and phenylalanine were impaired in Gnai3^-/-^ hepatocytes, we tested whether G_i_ signaling had an anti-autophagic role in BMDM [14]. We used BMDM prepared from Gnai3^-/-^, Gpsm1^-/-^, or wild type mice treated with PTX or not. We induced autophagy by rapamycin treatment, washed the cells, and added complete media (amino acids and serum). We tracked the changes in autophagic activity by LC3 immunoblotting (Figure 5 A-C) and autophagic vesicle visualization (Figure 5D). Unlike the studies with Gnai3^-/-^ primary hepatocytes, our results revealed no difference in the recovery from autophagy between BMDM derived from wild type mice and those from *Gnai3* ^*-/-*^ (Figure 5B), or *Gpsm1* ^*-/ -*^ (Figure 5C) mice. We found a similar result with PTX-exposed BMDM isolated from GFP-LC3 transgenic mice (Vural, A. and Kehrl, J.H., unpublished observations.) These results indicate that neither Gα_i_ nucleotide exchange nor the lack of Gα_i3_ or AGS3 impacted anti-autophagic activity in primary mouse macrophages. 

### The inhibition of Gα_i_ nucleotide exchange did not affect autophagic activity in transformed human macrophage cell line THP-1

**Figure 6:**
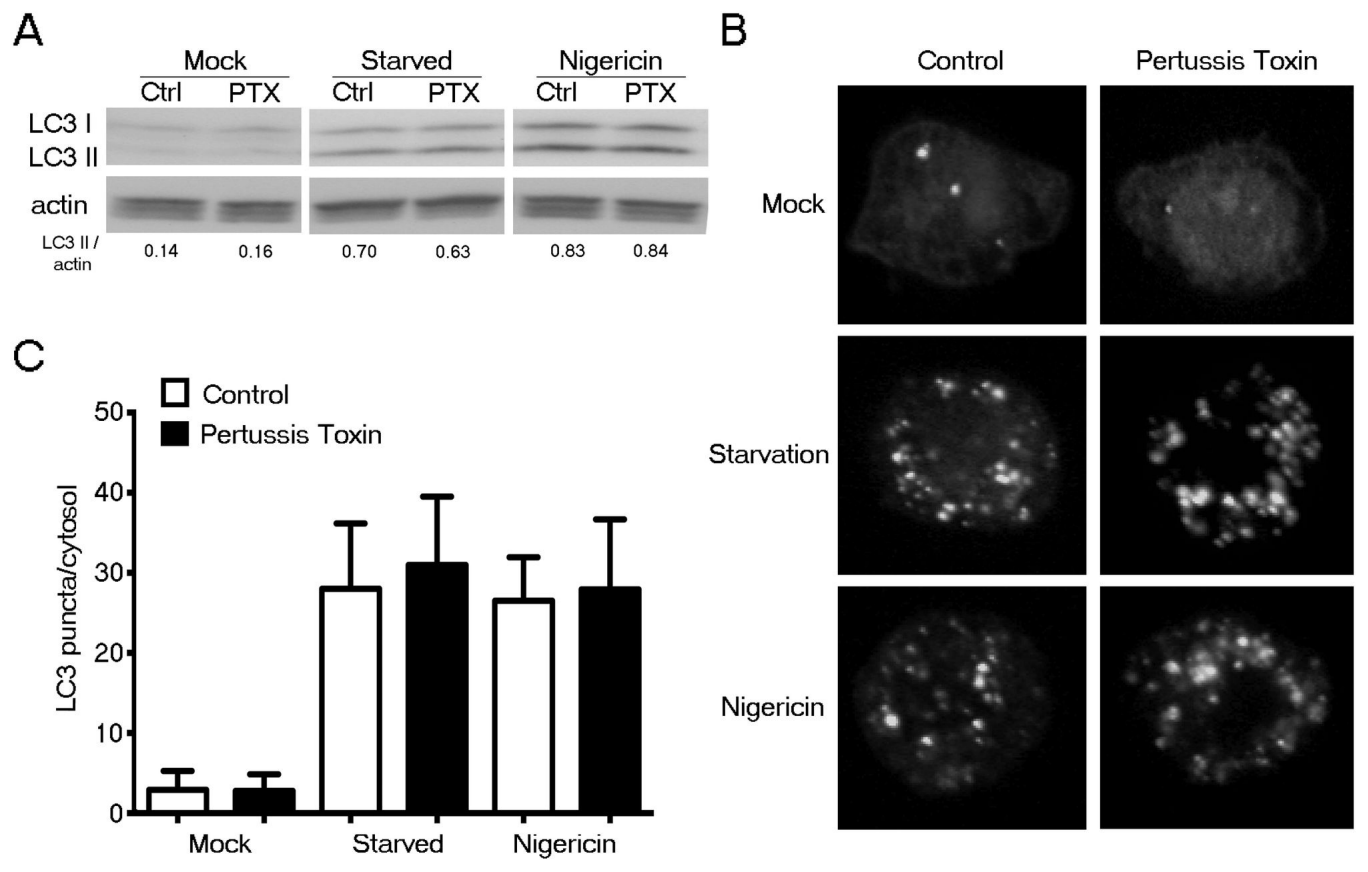
PTX treatment does not affect the autophagic activity in the human macrophage cell line THP-1. (*A*) Immunoblot analysis of THP-1 cell lysates untreated; or pre-exposed to PTX (200 ng/ml for 2h), either not further manipulated, starved (HBSS for 2h with 100 nM Bafilomycin A1), or treated with nigericin (4 µM for 2h) to assess LC3-II/actin band ratios. (*B*) Fluorescent microscopic images of LC3-GFP expression in a THP-1 GFP-LC3 stable cell line pre-exposed to PTX, or not and starved (HBSS for 2h with 100 nM Bafilomycin A1), or treated with nigericin (4 µM for 2h). Representative images are shown for each condition. (*C*) Quantification of GFP-LC3 dots in 50-100 cells for each condition from experiment shown in part B. Data represents the mean LC3 puncta per cytosol ± SEM from four independent experiments.

Our findings have relied on primary mouse cells while the other studies defining this pathway have used transformed human cell lines. To understand whether cellular transformation would alter the autophagy mechanism in macrophages, we used the transformed human macrophage cell line THP-1 that stably expressed GFP-LC3 as a platform to further assess the impact of G-protein mediated signaling on autophagy. We measured steady state, starvation- and nigericin-induced autophagic rates of THP-1 GFP-LC3 cells under normal and PTX treated conditions by LC3 immunoblotting and quantification of GFP-LC3 puncta (Figure 6 A-C). The results revealed no difference in autophagy levels between the two conditions showing that the inhibition of Gα_i_ nucleotide exchange does not affect autophagy in the transformed human cell line THP-1. This argues that there is an intrinsic difference between macrophages and other cell types, where the GTP binding status of Gα_i3_ has been shown to affect autophagic flux. 

## Discussion

There is growing evidence that autophagy plays key roles in innate and adaptive immunity, and in inflammation [34]. Autophagy facilitates the innate immune response by assisting in the capture and degradation of bacterial pathogens that invade cells [35]. Toll-like receptors (TLRs), potent inducers of innate immune responses, can enhance autophagy in dendritic cells and macrophages [36–38]. During the adaptive immune response, autophagy contributes to antigen presentation by major histocompatibility complex class II (MHCII) as well as autophagy impacts T and B lymphocyte homeostasis [35]. During inflammation, autophagy helps regulate the balance of inflammatory cytokines and metabolic responses [39,40]. As such, macrophages emerge as a paramount cell type for understanding the functional importance of autophagy. Delineating the regulatory mechanism that control the induction and the levels of autophagic sequestration in macrophages is of obvious interest, as they may be therapeutic targets for the manipulation of innate immunity and inflammation. We focused on a related set of proteins implicated in the control of autophagy in other cell types and assessed their importance in primary macrophages. However, despite their robust expression, the lack of Gα_i3_, AGS3, or RGS19 had little impact on the activation, induction, or sequestration of autophagosomes; on autophagic proteolysis of long-lived proteins; and on anti-autophagic activity in these cells. Contrasting with primary macrophages from Atg7^-/-^ mice and previous studies that examined cells from Atg5^-/-^ and Atg7^-/-^ mice, the macrophages from the Gnai3^-/-^, Gpsm1^-/-^, Rgs19^-/-^, and wild type mice expressed similar steady state levels of LC3, p62 and ubiquitinated proteins [41,42]. In addition, PTX treatment, which prevents Gα_i_ nucleotide exchange mediated by GPCRs and the non-receptor guanine nucleotide exchange factor Ric-8A, had no effect on basal or induced autophagosome formation [43]. 

A major caveat in the analysis of gene targeted mice is that related family members of the targeted gene can compensate for the loss of the targeted gene product. For several reasons, we do not think that gene compensation can reconcile our findings with the previous studies. First, the studies with Gα_i3_ and its regulators indicated that the response to manipulation of the nucleotide binding status of Gα_i3_ depended exclusively on Gα_i3_ and not on other Gα_i_ isoforms [9]. Like HT-29 cells and HeLa cells, primary mouse macrophages express Gα_i2_ and Gα_i3_. Thus, a compensatory increase in Gα_i2_ in the Gα_i3_ deficient macrophages would not explain the lack of a phenotype. Furthermore, immunoblotting macrophage cell lysates from Gnai3^-/-^ mice did not reveal any such increase in Gα_i2_ [14]. In addition, the results with the PTX treated wild type macrophages argue against an involvement of PTX sensitive G-proteins, which include Gα_i1-3_. The loss of AGS3 is unlikely to be compensated by AGS4, which lacks a TPR domain. The TPR domains of AGS3 and AGS5/LGN interact with a distinct set of proteins, thus it seems unlikely that the loss of AGS3 might be compensated for by an increase in LGN expression [44]. In contrast, the loss of RGS19 may well be compensated for by augmented expression of other RGS proteins although RGS19’s N-terminal cysteine string is unique among the RGS proteins [29]. The failure to observe any effect on basal autophagy or the induction of autophagosomes with macrophages from the different mutant mice together persuasively argues against a significant role for this regulatory pathway. 

Another explanation for our failure to find a role for Gα_i3_ and its regulators in the control of autophagy is related to differences in the cell types examined. We do not think it is related to specific protein expression levels in the different cell types as the BMDM expressed each of the proteins we examined. Also, it is unlikely related to a difference in Girdin/GIV expression as this gene is broadly expressed and found at levels above the general tissue mean in mouse macrophages (IMMGEN GeneSkyline/Constellation Symbol = Ccdc88a). In our study we focused on macrophages as a specific cell type using either primary mouse macrophages or a transformed human macrophage cell line. Perhaps macrophages lack an unknown, but essential protein, such that this signaling pathway simply does not impact the autophagy machinery in them. If this is the case, it reveals a tissue/cell type specific regulatory mechanism that impacts autophagy and indicates that care must be taken in drawing conclusions based on the study of a limited number of cell types. A recent study reveals such evidences regarding the differential regulation of autophagy in a tissue-dependent manner [45]. 

That Gα_i3_ has a non-redundant role in regulating autophagy in mouse liver is well supported by the studies of Gα_i3_ deficient mice where the anti-autophagic action of insulin and phenylalanine depended upon Gα_i3_ expression [14]. Furthermore, the subcellular localization of Gα_i3_ in starved hepatocytes changed following insulin or phenylalanine treatment moving from vesicular compartments to other intracellular sites. However, the mechanism underlying the failure of insulin or phenylalanine to reduce autophagy in the liver of the Gnai3^-/-^ mice remains unknown [46]. Gα_i3_ could well have a role in the insulin or phenylalanine upstream signaling pathway independent of any direct role in the regulation of autophagy at internal membranes. 

While we have not found a role for Gα_i3_ and its regulators in macrophage autophagy, most cells constantly assess their nutritional micro-environment by utilizing an array of promiscuous GPCRs that can sense amino acids, proteolytic degradation products, free fatty acids, and carbohydrates. Interference with some of those signaling pathways can trigger autophagy. For example, reducing the expression of T1R1/TIR3, a broadly expressed GPCR dimer that senses amino acid availability, altered the location of mTORC1, upregulated key amino acid transporters, and induced autophagy in both HeLa and H9C2 cells [47]. But it is unlikely that T1R1/T1R3 signals via a Gi-linked signaling pathway, but rather a Gq- or Gq family member or gustducin. It will be of interest to see if the T1R1/T1R3 dimer has a role in regulating autophagy in macrophages. Of note, since RGS19 has GTPase activating protein activity for Gq, its expression could alter signal transduction through such a pathway. 

In conclusion, although the results of our study seem to contradict previous findings, we are aware of the dynamic and multi-dimensional nature of autophagic process, which can be adjusted at multiple levels [33]. Our study relied upon mainly primary mouse and transformed human macrophages. The examination of LC3 processing and autophagy dependent long-lived protein degradation revealed that Gα_i3_, AGS3 and RGS19 have no essential role in the activation/induction of autophagosome formation, anti-autophagic activity and autophagic flux, respectively. Furthermore, no difference or inhibition of autophagosome clearance was detected as evidenced by similar levels of phosphatidyl ethanolamine (PE)-conjugated LC3-II and autophagosome numbers. Further studies assessing the mechanisms that control autophagy in macrophages are warranted because of their importance for innate and adaptive immunity. 
